# *N*-(4-Methyl­phenyl­sulfon­yl)maleamic acid

**DOI:** 10.1107/S1600536812032084

**Published:** 2012-07-18

**Authors:** H. Purandara, Sabine Foro, B. Thimme Gowda

**Affiliations:** a Department of Chemistry, Mangalore University, Mangalagangotri 574 199, Mangalore, India; b Institute of Materials Science, Darmstadt University of Technology, Petersenstrasse 23, D-64287 Darmstadt, Germany

## Abstract

In the title compound, C_11_H_11_NO_5_S, the dihedral angle between the benzene ring and the amide group is 76.88 (6)°. In the crystal, N—H⋯O(S) and O—H⋯O hydrogen bonds connect the mol­ecules into hydrogen-bonded layers perpendicular to the *a* axis.

## Related literature

For studies on the effects of substituents on the structures and other aspects of *N*-(ar­yl)-amides, see: Gowda *et al.* (2001 ▶); Shahwar *et al.* (2012 ▶), of *N*-(aryl­sulfon­yl)-succinamic acids, see: Purandara *et al.* (2012 ▶), of *N*-(ar­yl)-methane­sulfonamides, see: Gowda *et al.* (2007 ▶) and of *N*-chloro­aryl­sulfonamides, see: Gowda & Ramachandra (1989 ▶); Shetty & Gowda (2004 ▶).
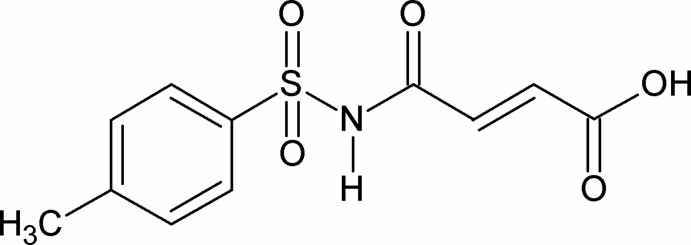


## Experimental

### 

#### Crystal data

C_11_H_11_NO_5_S
*M* *_r_* = 269.27
Monoclinic, 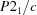

*a* = 10.2652 (6) Å
*b* = 11.9064 (7) Å
*c* = 10.2416 (6) Å
β = 108.403 (7)°
*V* = 1187.73 (12) Å^3^
*Z* = 4
Mo *K*α radiation
μ = 0.29 mm^−1^
*T* = 293 K
0.44 × 0.42 × 0.40 mm

#### Data collection

Oxford Diffraction Xcalibur diffractometer with a Sapphire CCD detector
Absorption correction: multi-scan (*CrysAlis RED*; Oxford Diffraction, 2009 ▶) *T*_min_ = 0.885, *T*_max_ = 0.894
4608 measured reflections
2424 independent reflections
2144 reflections with *I* > 2σ(*I*)
*R*_int_ = 0.010

#### Refinement

*R*[*F* ^2^ > 2σ(*F* ^2^)] = 0.031
*wR*(*F* ^2^) = 0.086
*S* = 1.07
2424 reflections
171 parameters
1 restraint
H atoms treated by a mixture of independent and constrained refinement
Δρ_max_ = 0.36 e Å^−3^
Δρ_min_ = −0.29 e Å^−3^

### 

Data collection: *CrysAlis CCD* (Oxford Diffraction, 2009 ▶); cell refinement: *CrysAlis CCD*; data reduction: *CrysAlis RED* (Oxford Diffraction, 2009 ▶); program(s) used to solve structure: *SHELXS97* (Sheldrick, 2008 ▶); program(s) used to refine structure: *SHELXL97* (Sheldrick, 2008 ▶); molecular graphics: *PLATON* (Spek, 2009 ▶); software used to prepare material for publication: *SHELXL97*.

## Supplementary Material

Crystal structure: contains datablock(s) I, global. DOI: 10.1107/S1600536812032084/zl2493sup1.cif

Structure factors: contains datablock(s) I. DOI: 10.1107/S1600536812032084/zl2493Isup2.hkl

Supplementary material file. DOI: 10.1107/S1600536812032084/zl2493Isup3.cml

Additional supplementary materials:  crystallographic information; 3D view; checkCIF report

## Figures and Tables

**Table 1:** Hydrogen-bond geometry (Å, °)

*D*—H⋯*A*	*D*—H	H⋯*A*	*D*⋯*A*	*D*—H⋯*A*
N1—H1*N*⋯O2^i^	0.81 (1)	2.17 (1)	2.9786 (16)	176 (2)
O5—H5*O*⋯O4^ii^	0.91 (2)	1.75 (2)	2.6589 (16)	176 (2)

Symmetry codes: (i) 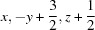; (ii) 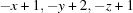.

**Fig. 1.**
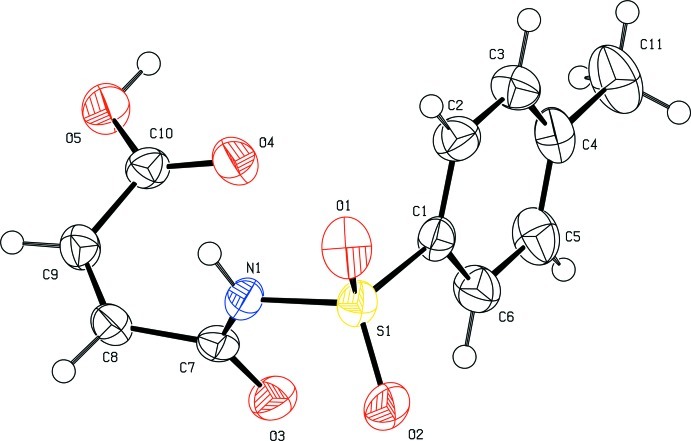
Molecular structure of the title compound, showing the atom labelling scheme with the displacement ellipsoids drawn at the 50% probability level.

**Fig. 2.**
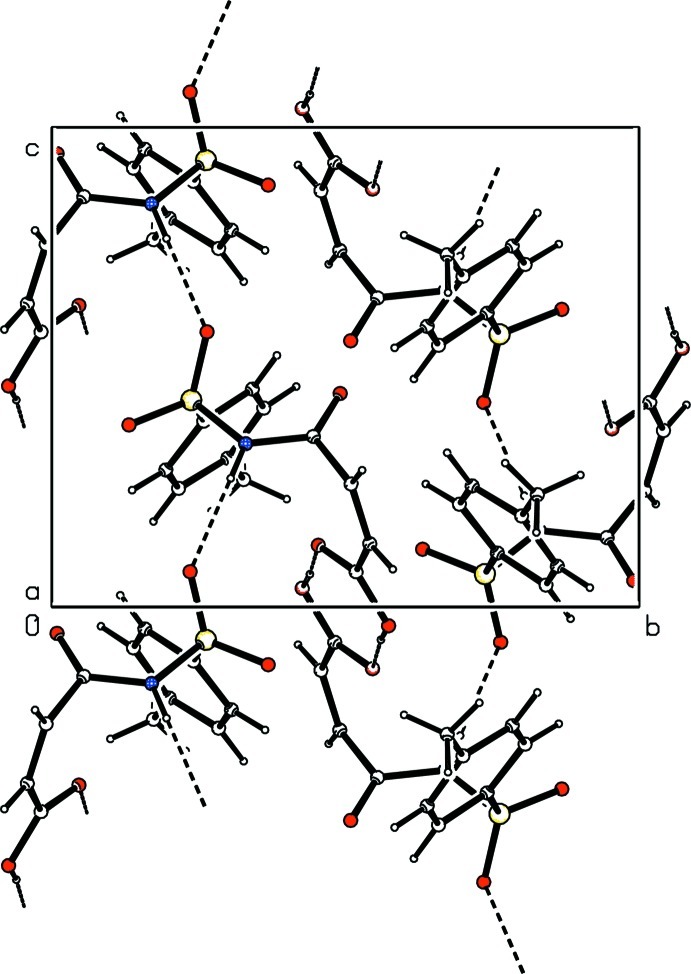
Molecular packing of the title compound with hydrogen bonding shown as dashed lines.

## supplementary crystallographic information

### Comment

As part of studies on the substituent effects on the structures and other aspects of *N*-(aryl)-amides (Gowda *et al.*, 2001; Shahwar *et al.*, 2012); *N*-(arylsulfonyl)-succinamic acids (Purandara *et al.*, 2012); *N*-(aryl)-methanesulfonamides (Gowda *et al.*, 2007) and *N*-chloroarylsulfonamides (Gowda & Ramachandra, 1989; Shetty & Gowda, 2004), in the present work, the crystal structure of *N*-(4-methylphenylsulfonyl)maleamic acid has been determined (Fig. 1).

The conformations of the N—H and C═O bonds in the amide segment are *anti* to each other and the amide C═O is *syn* to the adjacent C–H bond. Further, the amide C═O and the carboxyl C═O of the acid segment orient themselves almost perpendicular to each other, in contrast to the almost *anti* conformation observed between the amide C═O and the carboxyl C═O in *N*-(4-methylphenylsulfonyl)-succinamic acid (Purandara *et al.*, 2012). In the title compound, the C–H bonds on the –CH═ CH-group, adjacent to both the C═bonds, are *syn* to each other.

In the title compound, the C═O and O–H bonds of the acid group are in *syn* position to each other, similar to that observed in *N*-(4-methylphenylsulfonyl)-succinamic acid.

The molecule is bent at the S-atom with an C1–S1–N1–C7 torsion angle of 64.99 (13)°. Further, the dihedral angle between the phenyl ring and the amide group is 76.88 (6)°.

In the crystal, the intermolecular O—H···O and N—H···O(S) hydrogen bonds link the molecules into dimers and chains, respectively. The chains and dimers, in combination, form H-bonded layers perpendicular to the *a*-axis (Fig.2).

### Experimental

Maleic anhydride (0.015 mole) and triethylamine (0.01 mole) were added to a solution of *p*-toluenesulfonamide (0.01 mole) in dichloromethane. The reaction mixture was strirred for 18 h at room temperature and set aside for completion of the reaction. The reaction mixture was concentrated to dryness. The resultant title compound was washed with dilute HCl and then with water thoroughly, to remove the unreacted base and the maleic anhydride. It was recrystallized to constant melting point from ethyl acetate (144°C). The purity of the compound was checked and characterized by its IR spectrum. The characteristic absorptions observed are 3222.8 cm^-1^, 1700.7 cm^-1^, 1338.7 cm^-1^, 1257.4 cm^-1^, 1135.5 cm^-1^ and 851.3 cm^-1^ for the stretching bands of N–H, C═O, S═O asymmetric, C–N, S═O symmetric and C–S, respectively.

Prism like colorless single crystals used in the X-ray diffraction study were grown from ethyl acetate solution by slow evaporation of the solvent.

### Refinement

H atoms bonded to C were positioned with idealized geometry using a riding model with the aromatic C—H = 0.93 Å, methyl C—H = 0.96 Å. The amino H atom and the H atom of the OH group were freely refined with O—H = 0.91 (2) Å while the N—H distance later was restrained to 0.86 (2) Å, respectively. All H atoms were refined with isotropic displacement parameters set at 1.2 *U*_eq_(C-aromatic, N) and 1.5 *U*_eq_(*C*-methyl, *O*-hydroxyl) of the parent atom. The (4 0 0) and (2 1 0) reflections were probably affected by the beamstop and were omitted from the refinement.

### Crystal data

**Table:**

C_11_H_11_NO_5_S	*F*(000) = 560
*M**_r_* = 269.27	*D*_x_ = 1.506 Mg m^−^^3^
Monoclinic, *P*2_1_/*c*	Mo *K*α radiation, λ = 0.71073 Å
Hall symbol: -P 2ybc	Cell parameters from 2735 reflections
*a* = 10.2652 (6) Å	θ = 3.0–27.8°
*b* = 11.9064 (7) Å	µ = 0.29 mm^−^^1^
*c* = 10.2416 (6) Å	*T* = 293 K
β = 108.403 (7)°	Prism, colourless
*V* = 1187.73 (12) Å^3^	0.44 × 0.42 × 0.40 mm
*Z* = 4	

### Data collection

**Table:**

Oxford Diffraction Xcalibur diffractometer with a Sapphire CCD detector	2424 independent reflections
Radiation source: fine-focus sealed tube	2144 reflections with *I* > 2σ(*I*)
Graphite monochromator	*R*_int_ = 0.010
Rotation method data acquisition using ω and phi scans	θ_max_ = 26.4°, θ_min_ = 3.0°
Absorption correction: multi-scan (*CrysAlis RED*; Oxford Diffraction, 2009)	*h* = −12→12
*T*_min_ = 0.885, *T*_max_ = 0.894	*k* = −6→14
4608 measured reflections	*l* = −12→12

### Refinement

**Table:**

Refinement on *F*^2^	Secondary atom site location: difference Fourier map
Least-squares matrix: full	Hydrogen site location: inferred from neighbouring sites
*R*[*F*^2^ > 2σ(*F*^2^)] = 0.031	H atoms treated by a mixture of independent and constrained refinement
*wR*(*F*^2^) = 0.086	*w* = 1/[σ^2^(*F*_o_^2^) + (0.0449*P*)^2^ + 0.3604*P*] where *P* = (*F*_o_^2^ + 2*F*_c_^2^)/3
*S* = 1.07	(Δ/σ)_max_ = 0.001
2424 reflections	Δρ_max_ = 0.36 e Å^−^^3^
171 parameters	Δρ_min_ = −0.29 e Å^−^^3^
1 restraint	Extinction correction: *SHELXL97* (Sheldrick, 2008), Fc^*^=kFc[1+0.001xFc^2^λ^3^/sin(2θ)]^-1/4^
Primary atom site location: structure-invariant direct methods	Extinction coefficient: 0.029 (2)

### Special details

**Table:**

Experimental. CrysAlis RED (Oxford Diffraction, 2009) Empirical absorption correction using spherical harmonics, implemented in SCALE3 ABSPACK scaling algorithm.
Geometry. All e.s.d.'s (except the e.s.d. in the dihedral angle between two l.s. planes) are estimated using the full covariance matrix. The cell e.s.d.'s are taken into account individually in the estimation of e.s.d.'s in distances, angles and torsion angles; correlations between e.s.d.'s in cell parameters are only used when they are defined by crystal symmetry. An approximate (isotropic) treatment of cell e.s.d.'s is used for estimating e.s.d.'s involving l.s. planes.
Refinement. Refinement of *F*^2^ against ALL reflections. The weighted *R*-factor *wR* and goodness of fit *S* are based on *F*^2^, conventional *R*-factors *R* are based on *F*, with *F* set to zero for negative *F*^2^. The threshold expression of *F*^2^ > σ(*F*^2^) is used only for calculating *R*-factors(gt) *etc*. and is not relevant to the choice of reflections for refinement. *R*-factors based on *F*^2^ are statistically about twice as large as those based on *F*, and *R*- factors based on ALL data will be even larger.

### Fractional atomic coordinates and isotropic or equivalent isotropic displacement parameters (Å^2^)

**Table:**

	*x*	*y*	*z*	*U*_iso_*/*U*_eq_	
C1	0.36379 (14)	0.75860 (12)	0.11249 (15)	0.0323 (3)	
C2	0.45226 (16)	0.69418 (14)	0.21423 (16)	0.0408 (4)	
H2	0.4187	0.6370	0.2566	0.049*	
C3	0.59187 (17)	0.71591 (16)	0.25246 (17)	0.0463 (4)	
H3	0.6519	0.6731	0.3216	0.056*	
C4	0.64338 (16)	0.79985 (14)	0.18983 (18)	0.0431 (4)	
C5	0.55266 (18)	0.86083 (15)	0.0852 (2)	0.0482 (4)	
H5	0.5865	0.9161	0.0404	0.058*	
C6	0.41309 (17)	0.84149 (14)	0.04582 (18)	0.0432 (4)	
H6	0.3532	0.8835	−0.0243	0.052*	
C7	0.13459 (13)	0.94544 (13)	0.14452 (14)	0.0315 (3)	
C8	0.08229 (14)	1.00963 (12)	0.24353 (15)	0.0339 (3)	
H8	−0.0104	1.0285	0.2148	0.041*	
C9	0.15667 (15)	1.04155 (13)	0.36794 (15)	0.0364 (3)	
H9	0.1136	1.0807	0.4214	0.044*	
C10	0.30479 (15)	1.01820 (13)	0.42652 (14)	0.0343 (3)	
C11	0.79430 (18)	0.8267 (2)	0.2343 (3)	0.0666 (6)	
H11A	0.8436	0.7723	0.3007	0.100*	
H11B	0.8093	0.9002	0.2746	0.100*	
H11C	0.8263	0.8246	0.1558	0.100*	
N1	0.13089 (12)	0.83006 (11)	0.15952 (12)	0.0317 (3)	
H1N	0.1246 (18)	0.8042 (14)	0.2308 (15)	0.038*	
O1	0.16042 (12)	0.63163 (9)	0.12040 (14)	0.0492 (3)	
O2	0.12374 (12)	0.76432 (11)	−0.07348 (11)	0.0468 (3)	
O3	0.16830 (11)	0.99099 (10)	0.05504 (11)	0.0421 (3)	
O4	0.36494 (11)	0.95387 (10)	0.37140 (11)	0.0442 (3)	
O5	0.36415 (12)	1.07342 (11)	0.53916 (12)	0.0492 (3)	
H5O	0.456 (3)	1.060 (2)	0.571 (2)	0.074*	
S1	0.18624 (3)	0.73748 (3)	0.06857 (4)	0.03364 (14)	

### Atomic displacement parameters (Å^2^)

**Table:**

	*U*^11^	*U*^22^	*U*^33^	*U*^12^	*U*^13^	*U*^23^
C1	0.0310 (7)	0.0372 (8)	0.0306 (7)	−0.0021 (6)	0.0125 (6)	−0.0062 (6)
C2	0.0415 (8)	0.0442 (9)	0.0368 (8)	−0.0014 (7)	0.0126 (7)	0.0031 (7)
C3	0.0383 (8)	0.0549 (10)	0.0409 (9)	0.0062 (7)	0.0059 (7)	−0.0004 (7)
C4	0.0326 (8)	0.0456 (9)	0.0534 (10)	−0.0019 (7)	0.0168 (7)	−0.0155 (7)
C5	0.0415 (9)	0.0447 (9)	0.0656 (12)	−0.0033 (7)	0.0268 (8)	0.0035 (8)
C6	0.0381 (8)	0.0457 (9)	0.0482 (9)	0.0026 (7)	0.0170 (7)	0.0088 (7)
C7	0.0237 (6)	0.0401 (8)	0.0265 (7)	−0.0002 (5)	0.0020 (5)	−0.0001 (6)
C8	0.0276 (7)	0.0355 (7)	0.0374 (8)	0.0039 (6)	0.0082 (6)	0.0001 (6)
C9	0.0358 (7)	0.0366 (8)	0.0370 (8)	0.0045 (6)	0.0119 (6)	−0.0049 (6)
C10	0.0355 (7)	0.0351 (7)	0.0297 (7)	−0.0005 (6)	0.0064 (6)	−0.0034 (6)
C11	0.0335 (9)	0.0736 (14)	0.0916 (16)	−0.0064 (9)	0.0183 (10)	−0.0190 (12)
N1	0.0323 (6)	0.0375 (7)	0.0272 (6)	−0.0026 (5)	0.0121 (5)	−0.0023 (5)
O1	0.0494 (7)	0.0371 (6)	0.0666 (8)	−0.0124 (5)	0.0261 (6)	−0.0097 (5)
O2	0.0399 (6)	0.0664 (8)	0.0305 (6)	−0.0013 (5)	0.0060 (5)	−0.0148 (5)
O3	0.0444 (6)	0.0467 (7)	0.0363 (6)	0.0003 (5)	0.0143 (5)	0.0070 (5)
O4	0.0350 (6)	0.0494 (7)	0.0419 (6)	0.0057 (5)	0.0032 (5)	−0.0146 (5)
O5	0.0400 (6)	0.0629 (8)	0.0386 (6)	−0.0005 (6)	0.0037 (5)	−0.0203 (5)
S1	0.0305 (2)	0.0382 (2)	0.0325 (2)	−0.00642 (14)	0.01037 (15)	−0.00955 (14)

### Geometric parameters (Å, º)

**Table:**

C1—C2	1.379 (2)	C8—C9	1.318 (2)
C1—C6	1.384 (2)	C8—H8	0.9300
C1—S1	1.7520 (14)	C9—C10	1.474 (2)
C2—C3	1.386 (2)	C9—H9	0.9300
C2—H2	0.9300	C10—O4	1.2274 (18)
C3—C4	1.380 (2)	C10—O5	1.3002 (17)
C3—H3	0.9300	C11—H11A	0.9600
C4—C5	1.383 (3)	C11—H11B	0.9600
C4—C11	1.505 (2)	C11—H11C	0.9600
C5—C6	1.380 (2)	N1—S1	1.6551 (12)
C5—H5	0.9300	N1—H1N	0.813 (13)
C6—H6	0.9300	O1—S1	1.4246 (12)
C7—O3	1.2059 (17)	O2—S1	1.4286 (12)
C7—N1	1.384 (2)	O5—H5O	0.91 (2)
C7—C8	1.498 (2)		
			
C2—C1—C6	120.89 (14)	C7—C8—H8	117.3
C2—C1—S1	119.85 (12)	C8—C9—C10	123.19 (13)
C6—C1—S1	119.24 (12)	C8—C9—H9	118.4
C1—C2—C3	119.04 (15)	C10—C9—H9	118.4
C1—C2—H2	120.5	O4—C10—O5	124.10 (13)
C3—C2—H2	120.5	O4—C10—C9	122.31 (13)
C4—C3—C2	121.19 (15)	O5—C10—C9	113.58 (13)
C4—C3—H3	119.4	C4—C11—H11A	109.5
C2—C3—H3	119.4	C4—C11—H11B	109.5
C3—C4—C5	118.52 (14)	H11A—C11—H11B	109.5
C3—C4—C11	121.47 (17)	C4—C11—H11C	109.5
C5—C4—C11	120.01 (17)	H11A—C11—H11C	109.5
C6—C5—C4	121.46 (16)	H11B—C11—H11C	109.5
C6—C5—H5	119.3	C7—N1—S1	124.90 (10)
C4—C5—H5	119.3	C7—N1—H1N	119.2 (13)
C5—C6—C1	118.86 (15)	S1—N1—H1N	113.1 (13)
C5—C6—H6	120.6	C10—O5—H5O	111.8 (15)
C1—C6—H6	120.6	O1—S1—O2	120.05 (7)
O3—C7—N1	123.73 (14)	O1—S1—N1	104.01 (7)
O3—C7—C8	122.35 (14)	O2—S1—N1	107.49 (7)
N1—C7—C8	113.76 (12)	O1—S1—C1	109.40 (7)
C9—C8—C7	125.42 (13)	O2—S1—C1	109.01 (7)
C9—C8—H8	117.3	N1—S1—C1	105.91 (6)
			
C6—C1—C2—C3	2.1 (2)	C8—C9—C10—O4	−10.6 (3)
S1—C1—C2—C3	−176.73 (12)	C8—C9—C10—O5	168.24 (15)
C1—C2—C3—C4	−0.5 (3)	O3—C7—N1—S1	6.5 (2)
C2—C3—C4—C5	−1.4 (3)	C8—C7—N1—S1	−177.98 (9)
C2—C3—C4—C11	177.78 (16)	C7—N1—S1—O1	−179.73 (12)
C3—C4—C5—C6	1.9 (3)	C7—N1—S1—O2	−51.43 (13)
C11—C4—C5—C6	−177.29 (17)	C7—N1—S1—C1	64.99 (13)
C4—C5—C6—C1	−0.4 (3)	C2—C1—S1—O1	−16.74 (14)
C2—C1—C6—C5	−1.6 (2)	C6—C1—S1—O1	164.41 (12)
S1—C1—C6—C5	177.22 (13)	C2—C1—S1—O2	−149.80 (12)
O3—C7—C8—C9	−96.45 (19)	C6—C1—S1—O2	31.35 (14)
N1—C7—C8—C9	87.94 (18)	C2—C1—S1—N1	94.81 (13)
C7—C8—C9—C10	0.5 (3)	C6—C1—S1—N1	−84.04 (13)

### Hydrogen-bond geometry (Å, º)

**Table:**

*D*—H···*A*	*D*—H	H···*A*	*D*···*A*	*D*—H···*A*
N1—H1*N*···O2^i^	0.81 (1)	2.17 (1)	2.9786 (16)	176 (2)
O5—H5*O*···O4^ii^	0.91 (2)	1.75 (2)	2.6589 (16)	176 (2)

Symmetry codes: (i) *x*, −*y*+3/2, *z*+1/2; (ii) −*x*+1, −*y*+2, −*z*+1.
